# Hauntings, homeopathy, and the Hopkinsville Goblins: using pseudoscience to teach scientific thinking

**DOI:** 10.3389/fpsyg.2014.00336

**Published:** 2014-04-17

**Authors:** Rodney Schmaltz, Scott O. Lilienfeld

**Affiliations:** ^1^Department of Psychology, MacEwan UniversityEdmonton, AB, Canada; ^2^Department of Psychology, Emory UniversityAtlanta, GA, USA

**Keywords:** scientific thinking, skepticism, pseudoscience, teaching resources, introductory psychology

## Abstract

With access to information ever increasing, it is essential that students acquire the skills to distinguish fact from fiction. By incorporating examples of pseudoscience into lectures, instructors can provide students with the tools needed to understand the difference between scientific and pseudoscientific or paranormal claims. We discuss examples involving psychics, ghosts, aliens, and other phenomena in relation to scientific thinking. In light of research literature demonstrating that presenting and dispelling scientific misconceptions in the classroom is an effective means of countering non-scientific or pseudoscientific beliefs, we provide examples of pseudoscience that can be used to help students acquire healthy skepticism while avoiding cynicism.

From Dr. Oz promoting homeopathy to Deepak Chopra extolling the virtues of quantum healing, students are bombarded with questionable claims that require careful examination. Although students have access to more information than ever before, many do not possess the skills to distinguish good information from bad. Exacerbating this problem is the prevalence of pseudoscientific information available in the popular media, online, and even the classroom (Lilienfeld et al., 2004; Losh and Nzekwe, 2011; Novella, 2013). The purpose of this article is to provide examples that challenge students and provide instructors with tools to enhance scientific thinking. To do so, we describe how to distinguish science from pseudoscience, and provide several examples that can be used to promote scientific thinking. Specifically, we want to encourage students to employ *scientific skepticism*. Scientific skepticism means approaching claims with an open mind, and a willingness to accept only those claims that have survived scrutiny in rigorous scientific tests (Sagan, 1995). Skepticism differs from cynicism, which implies close-mindedness to novel claims. Through unique class demonstrations, assignments, and lecture material, instructors can use pseudoscience as a vehicle to engage students and foster scientific skepticism (see Stanovich, 2012 as a valuable resource).

Teaching scientific methods and the nature of science alone is not sufficient to help students distinguish science from pseudoscience. Data from educational psychology suggest that unless misconceptions are addressed explicitly in coursework, they will frequently persist (e.g., Winer et al., 2002). Overcoming students’ naïve scientific beliefs is a significant challenge for educators, as researchers have found that these beliefs can endure even after the acquisition of incompatible scientific theories (Shtulman and Valcarcel, 2012). In a survey of 10,000 American students over a 20-year period, there was only a modest decline in pseudoscientific beliefs following an undergraduate degree, even for students who had taken two or three science courses (Impey et al., 2012). At the same time, there is hope. Researchers have found that short-term skeptical thinking improves among students who have had direct exposure to the refutation of pseudoscientific claims (Kowalski and Taylor, 2009; Manza et al., 2010), although long-term follow-ups are needed to corroborate these findings. Incorporating examples of scientific misconceptions in lectures can be a valuable tool for science educators to help students overcome erroneous scientific beliefs and distinguish science from pseudoscience (see Lilienfeld et al., 2001 for a brief review of the literature).

## SCIENCE vs. PSEUDOSCIENCE

The distinction between science and pseudoscience is not clear-cut. The demarcation problem – the hoary question of how science differs from pseudoscience or non-science – can lead to fruitful class discussion (see Pigliucci and Boudry, 2013, for a thorough overview of the demarcation problem). Although this problem has not be definitively resolved, it can be helpful to provide a set of warning signs that indicate a claim may be pseudoscientific (for a review of the warning signs of pseudoscience, see Lilienfeld et al., 2012). From this perspective, science and pseudoscience differ in degree, not kind, but they often can be differentiated by means of a number of fallible, but useful, indicators (Stanovich, 2012). Some of these key warning signs are:

• *The use of psychobabble – words that sound scientific, but are used incorrectly, or in a misleading manner.* For example, “energy therapies” for psychological problems are often premised on biofeedback, meridian lines, quantum energies, and a host of other concepts that may sound impressive, but lack evidence.

• *A substantial reliance on anecdotal evidence.* Evidence for pseudoscience is typically anecdotal and consequently difficult to verify. For a class example, instructors may want to show students the Q-Ray bracelet website^1^ and read the many quotes submitted by Q-Ray users. Although the quotes sound compelling, there is no scientific evidence to support any claims attached to them. In fact, the Q-Ray company lost a lawsuit in 2011 and was ordered to refund over $11 million dollars to people who purchased a Q-Ray bracelet.

• *Extraordinary claims in the absence of extraordinary evidence* (Truzzi, 1978; Sagan, 1995). In pseudosciences, assertions are often highly implausible in light of existing knowledge yet are not backed by convincing evidence. For a class example, instructors may wish to describe how infomercials promoting Q-Ray bracelets state that the “bracelet rips [pain] right out of the body^2^.” and are “designed to optimize your natural positive energy^1^.”

• *Unfalsifiable claims* – Most pseudoscientific claims are incapable of being refuted in principle. For example, proponents of traditional Chinese medicine (TCM) believe the human body has an invisible energy force called Qi (Zollman and Vickers, 1999). Qi is a crucial component of TCM, even though it cannot be measured or tested scientifically.

• *An absence of connectivity to other research *(Stanovich, 2010). Connectivity refers to the extent to which assertions build on extant knowledge. For example, homeopathic practitioners state that homeopathic treatments become stronger as they become more dilute, and that water has memory. Both of these claims run counter to established scientific knowledge (Singh and Ernst, 2008).

• *Absence of adequate peer review.* Peer review is far from perfect, but it is a key safeguard against error. Instructors may wish to encourage students to contrast the claims advanced by the authors of peer-reviewed versus non-peer-reviewed articles.

• *Lack of self-correction.* Pseudosciences frequently persist despite refutation. Often, proponents of pseudoscience will use the idea that since the treatment or idea has been used for thousands of years it must be correct (e.g., astrology), an error often called the ad antiquetem fallacy (or, argument from antiquity).

One starting point to help students differentiate science from pseudoscience is to discuss the frequent misuse of quantum physics by proponents of the paranormal. *The Secret *(Byrne, 2006) may be one “secret” to starting students on their journey to become better scientific thinkers.**

## THE LAW OF ATTRACTION AND QUANTUM PHYSICS

Rhonda Byrne’s best-selling book *The Secret *(Byrne, 2006), based on the film of the same name, promotes the “law of attraction.” The basic tenet of this law is that like attracts like. This means that if we want something in our lives, we simply need to focus our thoughts on that object and it will come to us. Proponents of *The Secret* claim that the law of attraction works through sending out frequencies to the universe. This is explained through a misrepresentation of quantum physics and assorted psychobabble. A short video available on YouTube^3^ delineates the basic tenets of *The Secret*.

A useful class exercise is to allow students to watch the video and then attempt to devise an experiment to test the law of attraction. Students will start to grapple with issues of falsifiability and connectivity. *The Secret *is also an excellent tool to address such topics as psychobabble, how extraordinary claims require extraordinary evidence, and the need to investigate the reliability of the source when investigating claims (e.g., “Dr.” Joe Vitale has a Ph.D. from the online, unaccredited University of Metaphysics).

Instructors can provide a number of resources that fit the model of *The Secret*, such as QuantumMAN downloadable medicine^4^. QuantumMAN is an online resource whereby people can pay to download digital “medicine” that ostensibly cures everything from the common cold to malaria. Downloadable medicine supposedly “transfers from a remote quantum computer to your brain’s neural network for the benefits desired^4^.” The website is an ideal tool to discuss pseudoscience, as it encapsulates nearly all signs of pseudoscience.

## PSYCHICS AND SPOON BENDING

Students come into introductory psychology courses with many misconceptions about the field and science in general. One of the myths that many students hold is the idea that we only use 10% of our brain (for an overview of the myths of psychology, see Lilienfeld et al., 2010). Accessing the remaining 90% will supposedly lead to superior intelligence, and possibly even psychic abilities (Beyerstein, 1999). One of the proponents of the 10% myth is spoon-bending “mystifier” Uri Geller (Randi, 1982a). Geller rose to fame in the 1970s and was best known for his ability to bend metal by harnessing the remaining 90% of his brain.

A useful classroom demonstration is for the instructor to claim psychic abilities by bending spoons, supposedly using only the power of the mind. The method is simple, although it takes preparation. Before the class demonstration, instructors will need to take a cheap spoon and repeatedly bend it at the neck. Eventually, the neck of the spoon will become so weak that gently rubbing it will make the spoon bend or crack. This demonstration can generate a fruitful discussion on hypothesis generation. One hypothesis is that the instructor has psychic abilities, although students will quickly generate alternative hypotheses. Following this engaging demonstration, instructors may want to provide an overview of Uri Geller. Copious video footage of Geller in action is available online; such as Geller’s failure to show any psychic ability on *The Tonight Show*^5^.

Uri Geller is just one of many examples of purported psychics that can be a starting point for classroom discussion. Instructors can use videos or websites of celebrity psychics such as Sylvia Browne, John Edwards, or James van Praagh, and allow students to investigate the validity of their claims. Sylvia Browne, who passed away in 2013, made many appearances on such programs as the *Montel Williams Show*, *Larry King Live*, and other talk shows. In 2004, Browne appeared on the Montel Williams show and told the parents of Amanda Berry that their daughter had been murdered. In fact, Berry had been kidnapped in 2003, and escaped in 2013 in a high-profile story in Cleveland, Ohio. Sadly, Amanda’s mother died in 2006, believing that her daughter had been murdered.

Preeminent skeptic James Randi publicly challenged Sylvia Browne to prove her psychic ability. Randi, who wrote a book exposing Uri Geller (Randi, 1982a), is the founder of the James Randi Educational Foundation, which is dedicated to debunking pseudoscientific claims (Randi, 1982b). Randi, a former magician, offers a one million-dollar prize for anyone who can provide evidence of “paranormal, supernatural or occult power or events” in a scientifically controlled environment. Over the years, hundreds of people have tried, but no one has yet passed the preliminary tests. On an episode of *Larry King Live* in 2001, Browne agreed to take the million-dollar challenge, but refused to be tested after the program aired. As a class exercise, instructors can ask students to act as James Randi (see his appearance on CNN’s *Anderson Cooper360* discussing Sylvia Brown^6^), and design the test for Browne. Students should provide the evidence they would need before they would be willing to hand over the million dollars. This exercise helps students become engaged with research methodology, and highlights the type of extraordinary evidence needed to support extraordinary claims.

## ALIENS AND GOBLINS

From *The Day the Earth Stool Still* (1951) to *Dark Skies* (2013), aliens have been ubiquitous in popular culture. Alien abductions, invasions, or the mere existence of aliens provides a fascinating source of topics for classroom discussion. One example with which students may not be familiar is the curious case of the Hopkinsville Goblins (Nickell, 2006).

In 1955, 11 witnesses claimed that they were attacked by aliens, whom were approximately four feet tall, and bearing talons or claws. The aliens, or “goblins” as they were originally called, were silver, seemed to float or fly above the ground, and had thin legs. The goblins appeared shortly past 8:00 PM, and terrorized the family until midnight. At this point, some members of the family escaped and sought help from local authorities. Given the details and vivid eyewitness accounts, students may assume the case offers compelling evidence of alien visitation. Instructors should encourage students to consider what additional evidence would be required to accept this extraordinary claim, and to lay out plausible alternative explanations for the events.

The Hopkinsville entities have a decidedly earthly explanation. The “aliens” were in fact, Great Horned Owls, and the eyewitnesses were probably intoxicated during the “alien attack” (Davis and Bloecher, 1978). Students usually find the true story of the events amusing; and this example can lead naturally into a discussion on Area 51, the Greys, or other otherworldly interests (Nickell, 2012; Leman and Cinnirella, 2013).

## OTHER PSEUDOSCIENCE CLASSROOM ACTIVITIES

An informative class exercise is to allow students to hunt for examples of pseudoscience on their college or university campus. Upon return from their scavenger hunt, students can describe what they found, and why it could be considered pseudoscientific. Students are often surprised at how easy it is to find an example of pseudoscience on campus. Typically, they find books in the library, posters on campus promoting alternative health or study remedies, or advertisements in the school paper. The exercise drives home the point that students need to be skeptical thinkers, because questionable claims surround them.

Students can also be asked to locate examples of pseudoscience on television, online, or in books and magazines. One resource is *Most Haunted*, a British television program in which a team of investigators, including psychics and skeptics, examine haunted places. The methodology used by the team is far from rigorous, although students find the program entertaining. Instructors can ask students to imagine that they were part of the investigative team on *Most Haunted*, and then describe what would they do differently to determine whether ghosts are real.

The goal of using pseudoscientific examples is to create skeptical, not cynical, thinkers. As skeptical thinkers, students should be urged to remain open-minded. For example, they should not dismiss the existence of ghosts out of hand, but instead ask what evidence would convince them that spirits are among us. Students enjoy the challenge of creating their own ghost hunt, and this activity drives home key points in the discussion of scientific methodology. Although many shows are dedicated to hunting ghosts and monsters, *Most Haunted* is particularly useful, as the resident skeptic, Ciarian O’Keeffe, debunked one of the psychics on the program, Derek Acora. Dr. O’Keeffe suspected that Acora was being fed information prior to their investigations. To test this hypothesis, Dr. O’Keeffe created a story about “Kreed Kafer,” and left the information such that Acora had access to it. During the filming, Acora became “possessed” by the ghost of Kreed Kafer…which is an anagram of Derek Faker. The footage of the possession can be found on YouTube^7^.

Another task to help students understand the differences between science and pseudoscience is to ask the class to cooperate to create their own form of pseudoscience. Students can be quite creative as they try to “top” pseudoscientific ideas like QuantumMAN. One favorite was a project on past-life regression therapy – instead of past life, the students created future-life therapy. Through the magic of quantum mechanics, the supposed future-life therapist could look into the future for diseases, and administer treatment today with homeopathic remedies. Patients would know it was working if they did not suffer from the diseases that the therapist saw in the future. Brilliant!

These examples are only starting points. Although largely outside the scope of this paper, the topic of alternative medicine can also make for fruitful classroom discussion, especially because: (a) many students have had direct experience with it and (b) there is serious question regarding whether any form of alternative medicine works better than placebo (Bausell, 2007). For instance, hundreds of studies have investigated homeopathy, and although a few meta-analyses show effects beyond a placebo (e.g., Kleijnen et al., 1991; Linde et al., 1997; Cucherat et al., 2000), the methodological quality of the research in these meta-analyses is poor. To explore these points, instructors can ask students to conduct a literature review in peer-reviewed journals on homeopathy or related topics, and contrast it with what is found in non-peer reviewed sources. Students will find that the evidence from reputable journals indicates that homeopathic remedies do not work (Singh and Ernst, 2008; Shapiro, 2009; Ernst, 2010). A comprehensive list of other questionable alternative remedies, such as acupuncture, energy therapy, and rebirthing, can be found at www.quackwatch.com.

We further encourage instructors to explore resources such as the Penn & Teller program *Bullshit* (although as the name suggests, the language is coarse, and the tone can at times be mean-spirited) and comedian-songwriter Tim Minchin’s amusing videos on YouTube on scientific thinking and skepticism (such as his ode to Oberg’s Dictum, or the notion that we should keep an open mind but not so open that our “brains fall out,” at http://youtu.be/bBUc_kATGgg). Prominent skeptics such as the late Carl Sagan, Michael Shermer, Phil Plaitt, and Richard Wiseman all have many resources available online, and are well worth exploring. By capitalizing on these and other excellent online sources, instructors can persuade students that scientific thinking is not only invaluable as a means of evaluating claims in everyday life, but immensely fun as well.

## A WARNING

As a caveat to instructors, research suggests that the use of pseudoscientific examples enhances scientific thinking, but only if framed correctly. The presentation of pseudoscience in a class that typically focuses on science can occasionally confuse students and even lead to “backfire effects” (in which they come to view the unsupported claim as well-supported), as some students may remember the pseudoscientific example, but forget that it is discredited (Lewandowsky et al., 2012). Instructors need to ensure that students understand the examples are pseudoscientific by referring back to the signs of pseudoscience within the discussion of each pseudoscientific example. For additional strategies to avoid student confusion, see Lewandowsky et al.’s (2012) review of the misinformation effect and how to correct for it.

## CONCLUDING THOUGHTS

Especially in today’s world of 24/7 information and misinformation, students need to be able to evaluate extraordinary claims of many kinds. Fortunately, by directly addressing and then refuting non-scientific claims, science educators can dispel pseudoscience and promote scientific skepticism, while avoiding the unhealthy extremes of either uncritical acceptance or cynicism. In this way, science instructors can help students to become more thoughtful and discerning consumers of evidence, not only in the classroom, but in daily life.

## Conflict of Interest Statement

The authors declare that the research was conducted in the absence of any commercial or financial relationships that could be construed as a potential conflict of interest.

## Footnotes

1 http://www.qray.com

2 http://www.quackwatch.org

3 http://youtu.be/zdtqLNeK6Ww

4 http://www.quantummansite.com

5 http://youtu.be/lTn0t_7pGZo

6 http://youtu.be/YuPadpaTwKY

7 http://youtu.be/ZbF_l5nwmGs
