# Corrigendum: Hauntings, homeopathy, and the Hopkinsville Goblins: using pseudoscience to teach scientific thinking

**DOI:** 10.3389/fpsyg.2017.01982

**Published:** 2017-11-07

**Authors:** Rodney Schmaltz, Scott O. Lilienfeld

**Affiliations:** ^1^Department of Psychology, MacEwan University, Edmonton, AB, Canada; ^2^Department of Psychology, Emory University, Atlanta, GA, United States

**Keywords:** scientific thinking, skepticism, pseudoscience, teaching resources, introductory psychology

In the original article, there was an incorrect citation. In the third paragraph under the heading, “Aliens and Goblins”, the reference to Davis and Bloecher (1978) is incorrect. The reference should be Nickell (2006).

A correction has been made to this section:

The Hopkinsville entities have a decidedly earthly explanation. It is plausible, if not likely, that the “aliens” were Great Horned Owls, and there is some evidence that the eyewitnesses may have been intoxicated during the “alien attack” (Nickell, 2006). Students usually find the true story of the events amusing; and this example can lead naturally into a discussion on Area 51, the Greys, or other otherworldly interests (Nickell, 2012; Leman and Cinnirella, 2013). The authors apologize for this error and state that this does not change the scientific conclusions of the article in any way.

## Conflict of interest statement

The authors declare that the research was conducted in the absence of any commercial or financial relationships that could be construed as a potential conflict of interest.

